# MMP9 Expression Correlates With Cisplatin Resistance in Small Cell Lung Cancer Patients

**DOI:** 10.3389/fphar.2022.868203

**Published:** 2022-04-01

**Authors:** Longqiu Wu, Xiangcai Wang, Xin He, Qiang Li, Qian Hua, Rongrong Liu, Zhengang Qiu

**Affiliations:** ^1^ Department of Oncology, The First Affiliated Hospital of Gannan Medical University, Ganzhou, China; ^2^ Department of Neurology, Ganzhou People’s Hospital, Ganzhou, China; ^3^ Department of Neurology, The First Affiliated Hospital of Gannan Medical University, Ganzhou, China

**Keywords:** small lung cell cancer, cisplatin, MMP9, survival, resistance

## Abstract

**Background:** Cisplatin is the basis of the primary treatment for SCLC chemotherapy. However, the limited objective response rate and definite drug resistance greatly restrict the clinical potential and therapeutic benefits of cisplatin use. Therefore, it is essential to identify biomarkers that can discern the sensitivity of SCLC patients to cisplatin treatment.

**Methods:** We collected two SCLC cohorts treated with cisplatin that included mutation data, prognosis data and expression data. The sensitivity of cisplatin was evaluated by the pRRophetic algorithm. MCPcounter, quanTIseq, and xCell algorithms were used to evaluate immune cell score. GSEA and ssGSEA algorithms were used to calculate immune-related pathway scores. Univariate and multivariate Cox regression models were employed, and survival analysis was used to evaluate the prognostic value of the candidate genes.

**Results:** MMP9-High is related to improved clinical prognoses of patients with SCLC (HR = 0.425, *p* = 0.0085; HR = 0.365, *p* = 0.0219). Multivariate results showed that MMP-High could be used as an independent predictor of the prognosis of SCLC after cisplatin treatment (HR = 0.216, *p* = 0.00153; HR = 0.352; *p* = 0.0199). In addition, MMP9-High displayed a significantly lower IC50 value of cisplatin and higher immunogenicity than MMP9-Low SCLC. Compared with MMP9-Low SCLC, MMP9-High included significantly increased levels of T-cells, cytoxic lymphocytes, B-cells, NK-cells, and dense cells (DCS). Similarly, the activity of cytokine binding, B-cell, NK-cell mediated immune response chemokine binding, and antigen presentation pathways in MMP9-High was significantly higher than that in MMP9-Low.

**Conclusion:** In this study, we identified that MMP9-High could be potentially considered a novel biomarker used to ascertain the improved prognosis of SCLC patients after cisplatin treatment. Furthermore, we indicated that the tumor immune microenvironment of MMP9-High SCLC is mainly characterized by a large number of infiltrated activated immune cells as well as activated immune-related pathways.

## Introduction

Small cell lung cancer (SCLC), which accounts for 13–15% of lung cancer, is a subtype of lung cancer known to exhibit high malignancy and poor prognosis (Sabari et al., 2017; Qiu et al., 2019; Luo et al., 2019). The median survival time of SCLC patients is only 8–13 months, and the 5-year survival rate is 1–5% (de Hoyos and DeCamp, 2014; Carter et al., 2014). Chemotherapy for SCLC is comprised of a combination approach that includes platinum-based antineoplastic drugs commonly used in the treatment of various cancers. Cisplatin a well-known, effective, and widely used first-line drug with an objective response rate (ORR) of 50–60% (Horn et al., 2018). However, almost all patients diagnosed with SCLC will inevitably present with drug resistance and tumor recurrence. Studies have shown that the functional mechanism of cisplatin resistance occurs as a result of structural changes to DNA or cytoplasm, the abnormality of DNA damage repair, the change of signaling triggered by molecular damage caused by cisplatin, and the change of compensatory survival signal (Galluzzi et al., 2014; Inoue et al., 2014; Song et al., 2015). However, the lack of biomarkers used to identify cisplatin sensitivity in SCLC population is a large clinical detriment. Therefore, it is of great importance to find biomarkers that pertain to the sensitivity of SCLC patients undergoing cisplatin treatment.

Evidence has recognized the close relationship between tumor immune microenvironment (TIME) and chemotherapeutic drugs. Tumor-associated macrophages (TAMs), tumor-associated neutrophils (TANs), myeloid-derived suppressor cells (MDSCs), regulatory T-cells, Immunosuppressive cells such as T-regulatory cells (Tregs), and regulatory B-cells (Bregs) can not only directly inhibit killer cells such as CTL and NK, but also interact with each other, enhancing the effect of inhibitory factors and facilitating the recruitment of more immunosuppressive cells. This process enables tumor cells to achieve immune escape (Lin et al., 2019). Additionally, TAMs are known to secrete TGF-β1, which results in the up-regulation of Gfi-1 expression in tumor cells. Gfi-1 expression in the promoter region effectively inhibits the expression of CTGF and HMGB1, which accordingly reduce the sensitivity of tumor cells to gemcitabine (Xian et al., 2017). TAMs expressed IGF act on the IGF1 receptor to promote chemotherapy resistance of gemcitabine and albumin-bound paclitaxel (Ireland et al., 2016). In addition, the study showed that during the chemotherapy of gemcitabine, TAMs, G-MDSCs, Tregs and T-cells decreased, and CTL increased (Mitchem et al., 2013; Eriksson et al., 2016). Gemcitabine promoted the up regulation of HLA-DR, CD40, CCR7 and the down regulation of CD163 and cd206, and induced M1 polarization (Di Caro et al., 2016). In conclusion, chemotherapy drugs can act on TIME, and TIME can also affect the effect of chemotherapy drugs.

Matrix metalloproteinases (MMPs) play an important role in tumorigenesis, development, invasion and metastasis. MMP9 is the largest molecular weighted enzyme in the MMP family. The function of MMP9 is closely related to tumor invasion and metastasis through its functional degradation of type IV and V collagen and gelatin (Mondal et al., 2020). Studies have shown that MMP9 expression is regulated in ovarian cancer, cervical cancer, non-small cell lung cancer and breast cancer, all of which demonstrate a close relationship to cisplatin sensitivity (Rauvala et al., 2006; Braicu et al., 2014; Qiao et al., 2020; Makhoul et al., 2016). In addition, Li et al. found evidence that MMP9 can regulate the biological function of monocytes (Zhou et al., 2012; Li et al., 2021; Xu et al., 2021). Furthermore, Xu et al. found that the high MMP9 group was significantly enriched in the immune response pathway and cytokine production pathway (Xu et al., 2021). However, at present, the expression of MMP9 and cisplatin sensitivity in SCLC patients has not been clarified and the associated relationship with the tumor immune microenvironment is not clear. Therefore, in this study, we aimed to explore the relationship between MMP9 expression and cisplatin sensitivity as well as elucidate the TIME in SCLC, so as to provide a theoretical basis for the precise treatment of SCLC and improve the clinical benefits to patients.

## Methods

### SCLC Cohort

**FIGURE 1:**
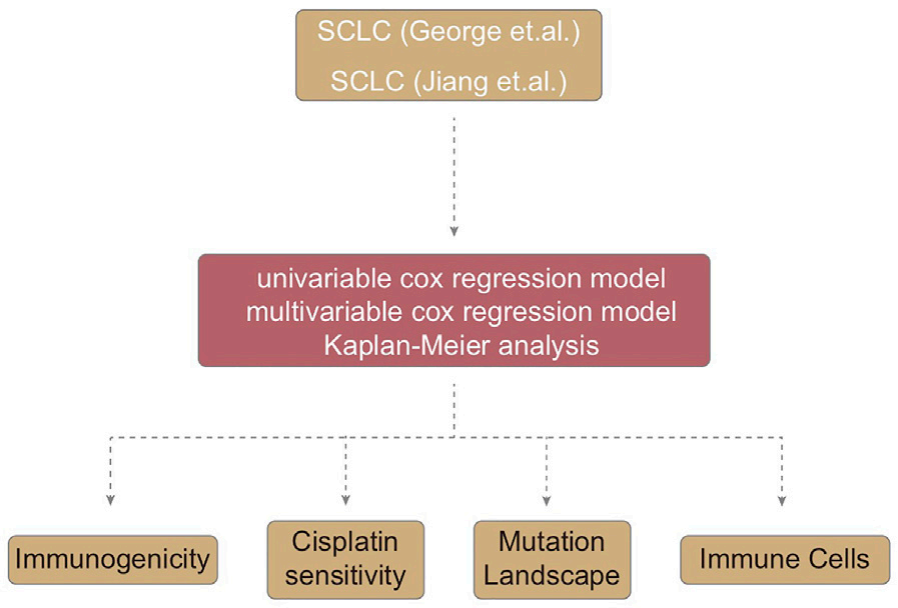
The comprehensive study design.

We collected two published SCLC cohorts from the gene expression omnibus (GEO) database (Clough and Barrett, 2016), namely SCLC (George et al., 2015) and SCLC (Jiang et al., 2016). In this study, the inclusion criteria for SCLC patients were that the included SCLC patients must have survival data, expression data, and mutation data. According to the above inclusion criteria, a total of 68 SCLC patients were recorded from paper 1 (Supplementary Table S1) and 48 SCLC patients from paper 2 (Supplementary Table S2). We filtered the mutation data of the two SCLC cohorts according to the definition and type of non-synonymous mutation in a maftools R package (Mayakonda et al., 2018). The non-synonymous mutation data obtained after filtering was then used for subsequent analyses. The analysis process of this study is detailed in Figure 1.

### Prediction of Cisplatin Sensitivity

We used the pRRophetic algorithm (Geeleher et al., 2014) to predict the IC50 value of cisplatin by constructing a relevant ridge regression model with a GDSC cell line expression profile as a training set, and the SCLC cohort as validation set. According to the median value of MMP9 expression, SCLC patients were divided into MMP9-High and MMP9-Low categories. The IC50 values of cisplatin between the two groups were analyzed using the Mann Whitney *U* test. Further, a Spearman correlation test was used to analyze the expression of MMP9 and the related IC50 value of cisplatin.

### Analysis of Tumor Immune Microenvironment Infiltration

MCPcounter, quanTIseq and xCell algorithms were used to analyze the expression profile data of SCLC and obtain the overall score of immune cells (Becht et al., 2016; Aran et al., 2017; Plattner et al., 2020). GSEA was used to analyze the difference of signal pathway activity between MMP9-High and MMP9-Low classes (Reimand et al., 2019). ssGSEA (Hänzelmann et al., 2013) was used to evaluate the signal pathway activity of each SCLC patient in the c2 and c5 pathway sets according to the MsigDB database (Liberzon et al., 2011).

### The Predictive Value of MMP9 in an Immunotherapy Cohort

We verified the prognostic value of MMP9 expression in the NSCLC cohort receiving ICIs treatment using a CAMOIP webpage tool (Lin et al., 2021a).

### Immunogenicity Analysis

The mutation data and expression data of the TCGA cohort were downloaded using the TCGAbiolinks R package. The data of tumor mutation burden (TMB) and neoantigen loads (NALs) of the TCGA cohort were obtained from published literature (Thorsson et al., 2018).

### Immunohistochemistry

Tissue samples were deparaffinized and rehydrated. After treatment with endogenous peroxidase blocking solution, they were treated with specific antibodies against MMP9 (ab76003, Abcam), overnight at 4°C. After they were washed with PBS, the samples were treated with horseradish peroxidase-conjugated anti-rabbit IgG (SV0002, Boster, Wuhan, China) and then stained with diaminobenzidine (DAB). All results were assessed by two pathologists. Expression levels were scored by multiplying the percentage of positive cells by the staining intensity. The positivity percentage was scored as 0 if <5% (negative), 1 if 5–30% (sporadic), 2 if 30–70% (focal) and 3 if 70% (diffuse) of the cells were stained; and staining intensity was scored as 0 for no staining, 1 for weak to moderate staining and 2 for strong staining. A score of ≥2 was regarded as ‘high’ and the score of <2 is regarded as “low” in immunohistochemical staining.

### Cell Counting Kit-8 Assay

Cells were cultured at 5 × 10^3^ cells per well in a 96-well plate with cytotoxic drugs for 24 h. Cytotoxic drugs (cisplatin and etoposide) were diluted to obtain different concentration gradients. Absorbance was detected at 450 nm after treatment with 10 μl CCK-8 reagent (Dojindo, Kumamoto) for 4 h. The experiments were performed in five replicate wells per sample and the assays were conducted in triplicate.

### Quantitative Real-Time PCR

Total RNA was isolated using Trizol reagent (Invitrogen, United States), according to the manufacturer’s instructions. The quantity and purity of the total RNA was measured using the Nanodrop^®^ ND1000 (Thermo Fisher) and the Agilent Bioanalyzer. Reverse transcription was performed with 2 μg of total RNA using M-MLV reverse transcriptase (Accurate Biology, AG11728) according to the manufacturer’s recommendations. Quantitative PCR was performed using CFX96 Touch Real-Time PCR Detection Instrument (BioRad, United States). Reactions were performed using SYBR^®^ Green Premix Pro Taq HS qPCR Kit (ROX Plus) (AG11718). Values were normalized to GAPDH via the 2^−ΔΔCt^ method.

### Statistical Analysis

The statistical results of KM survival analysis were obtained by a log rank test, and the visual results of KM survival analyses were obtained by survivor and survminer. Univariate COX and multivariate COX models were used to verify whether MMP9 can be used as an independent predictor of the prognosis of SCLC patients treated with cisplatin. The different analysis of continuity variables between MMP9-High and MMP9-Low groups was completed by the Mann Whitney *U* test. In this study, all the analysis is based on R software. The *p* value is bilateral, and *p* < 0.05 is regarded as statistically significant.

## Results

### MMP9 is an Independent Predictor of the Prognosis of SCLC Treated With Cisplatin

**FIGURE 2:**
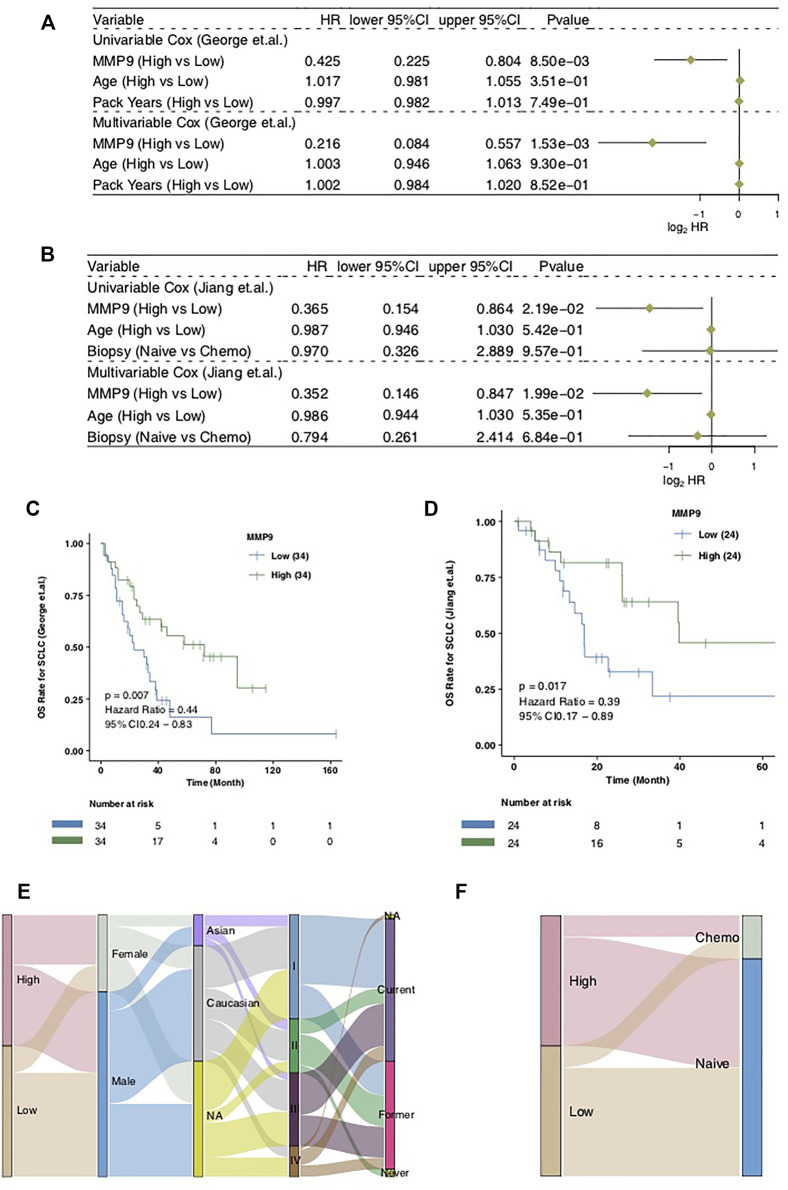
The prognostic value of MMP9. The univariate and multivariate COX regression model of the SCLC (George et al.) **(A)** and the SCLC (Jiang et al.) **(B)**. Kaplan-Meier curves comparing the progress free survival (PFS) of patients with MMP9-High and patients with MMP9-Low in the SCLC (George et al.) **(C)** and the SCLC (Jiang et al.) **(D)**. A Sankey diagram visualizing the clinical characteristics between MMP9-High and MMP9-Low patients in the SCLC (George et al.) **(E)** and the SCLC (Jiang et al.) **(F)**.

In order to explore the influence of MMP9 on the prognosis of patients with SCLC treated with cisplatin, we used the univariate COX regression model and multivariate COX regression model to evaluate the SCLC cohort, including SCLC (George et al.) and SCLC (Jiang et al.). In SCLC (George et al.), we found that only MMP9-High was related to an improved clinical prognosis of patients with SCLC, while common clinical factors were not relevant in the prognosis of patients (Figure 2A; HR = 0.425; *p* = 0.0085). Multivariate results showed that MMP-High could be used as an independent predictor of prognosis of SCLC after cisplatin treatment (Figure 2A; HR = 0.216; *p* = 0.00153). Then, univariate COX and multivariate COX regression models were also applied to SCLC (Jiang et al.), and the results showed that MMP9-High was not only related to significantly prolonged clinical prognosis time (Figure 2B; HR = 0.365; *p* = 0.0219) but it can also be used as an independent predictor (Figure 2B; HR = 0.352; *p* = 0.0199). The KM curve shows that MMP9-High is related to significantly prolonged OS in patients with SCLC (Figure 2C: log-rank *p* = 0.007; HR = 0.44; Figure 2D: log-rank *p* = 0.017; HR = 0.39). We used a Sankey diagram to visualize the clinical features of two SCLC cohorts one by one (Figures 2E,F).

### MMP9 is Related to Cisplatin Sensitivity and Immunogenicity

**FIGURE 3:**
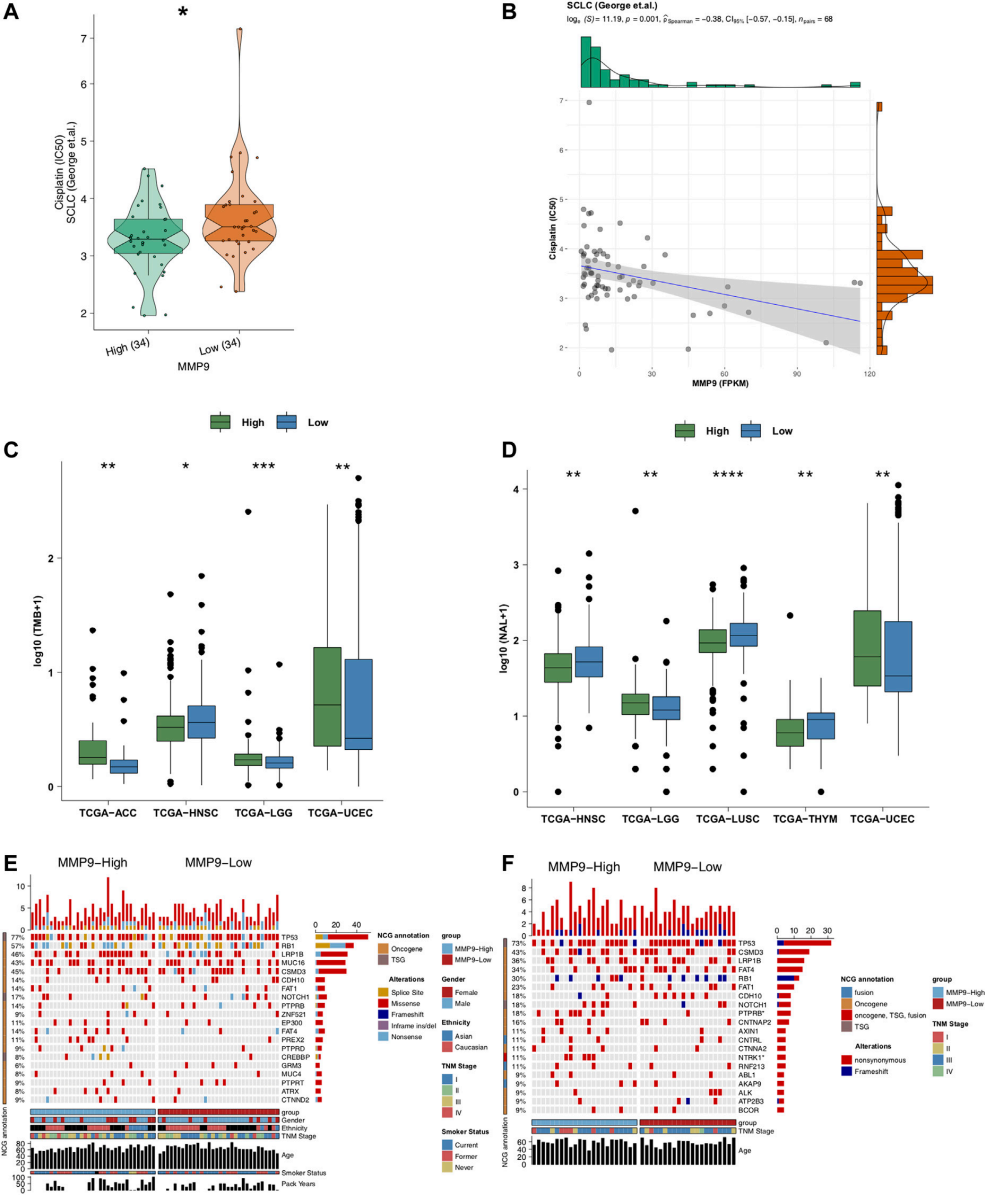
The association between MMP9, immunogenicity, and cisplatin. **(A)** Comparison of IC50 values of cisplatin between MMP9-High and MMP9-Low tumors. **(B)** The association between the IC50 value of cisplatin and the expression of MMP9. **(C)** Comparison of the tumor mutation burden between MMP9-High and MMP9-Low tumors. **(D)** Comparison of neoantigen loads (NALs) between MMP9-High and MMP9-Low tumors. The top 20 mutated driver genes in the SCLC (George et al.) **(E)** and SCLC (Jiang et al.) **(F)**. (*p < 0.05; **p < 0.01; ***p < 0.001; ****p < 0.0001; Mann-Whitney *U* test).

We employed the pRRophetic algorithm to predict the cisplatin sensitivity of each SCLC patient to obtain an IC50 value. We found that MMP9-High had significantly lower IC50 values of cisplatin than MMP9-Low SCLC (Figure 3A; *p* < 0.05). Similarly, the expression of MMP9 was negatively correlated with the IC50 value of cisplatin (Figure 3B, *p* = 0.001; R = −0.38; method: spearman). We found that MMP9-High had significantly higher TMB than MMP9-Low (Figure 3C; All *p* < 0.05) and NALs (Figure 3D; all *p* < 0.05). Figure 3E shows that for SCLC (George et al.), the type and mutation frequency of the driver genes in the top 20 mutation frequencies in the cohort. The results showed that there was no significant difference between the driver genes in the top 20 mutation frequencies of MMP9-High and MMP9-Low. In SCLC (Jiang et al.), the mutation frequency of MMP9-High is significantly higher than that of MMP9-Low in PTPRB and NTRK (Figure 3F). Supplementary Figures S1A,B shows the mutual exclusion and co-occurrence of the top 20 driving mutations in MMP9-High and MMP9-Low, respectively.

### MMP9 is Related to Activated Immune Cells

**FIGURE 4:**
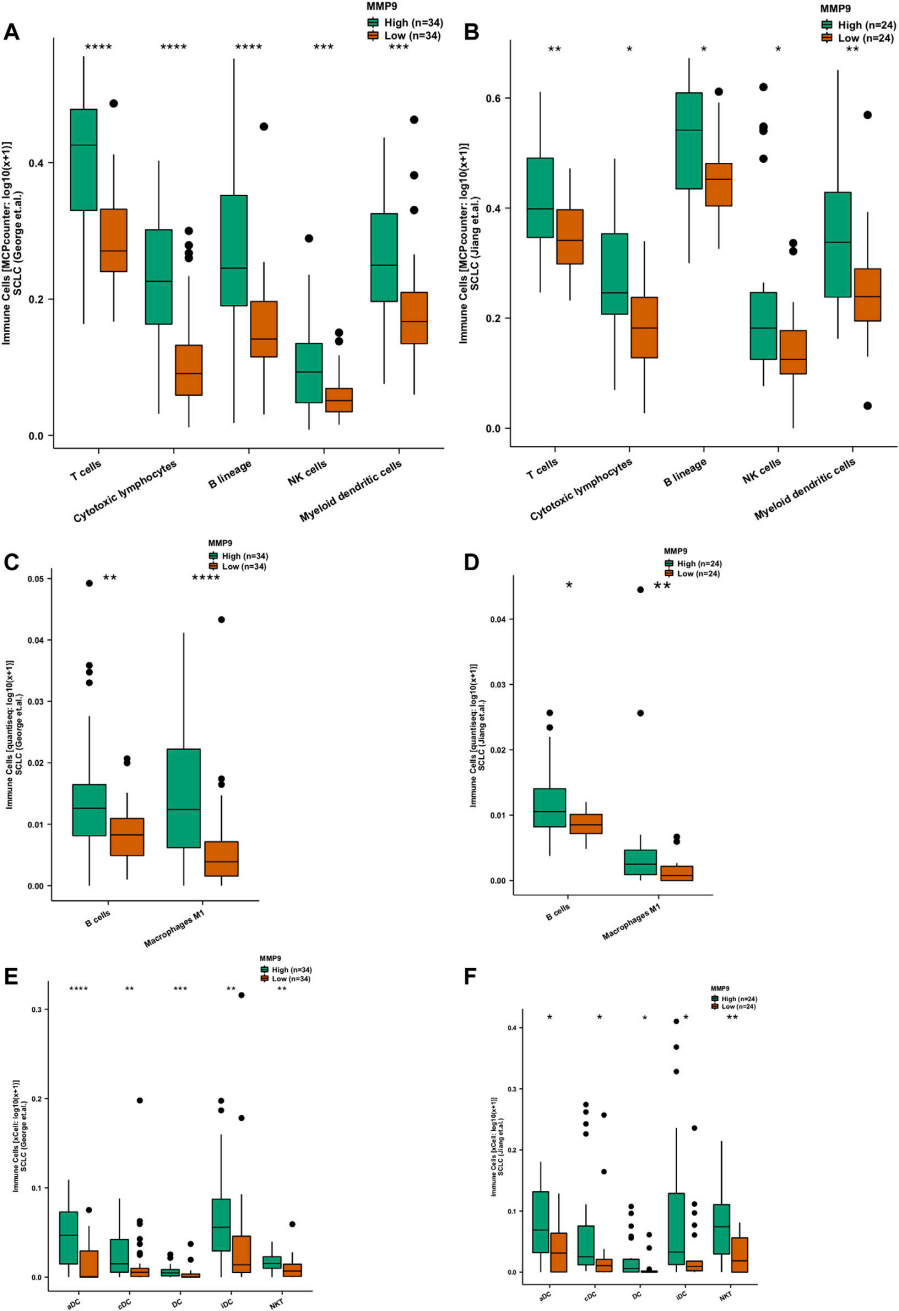
The association between MMP9 and immune cells. Comparison of immune cells estimated according to MCPcounter between MMP9-High and MMP9-Low tumors in the SCLC (George et al.) **(A)** and the SCLC (Jiang et al.) **(B)**. Comparison of immune cells estimated by quanTIseq between MMP9-High and MMP9-Low tumors in the SCLC (George et al.) **(C)** and the SCLC (Jiang et al.) **(D)**. Comparison of immune cells estimated by xCell between MMP9-High and MMP9-Low tumors in the SCLC (George et al.) **(E)** and the SCLC (Jiang et al.) **(F)**. (*p < 0.05; **p < 0.01; ***p < 0.001; ****p < 0.0001; Mann-Whitney *U* test).

Under the MCPcounter algorithm, whether in SCLC (George et al.) or SCLC (Jiang et al.), we found that the TIME when MMP9-High was higher than MMP9-Low was significantly infiltrated with t-cells, cytoxic lymphocytes, B-cells, NK cells, and dense cells (DCS) (Figures 4A,B; all *p* < 0.05). Under the quanTIseq algorithm, we found that MMP9-High has significantly increased B cells and m1 macrophages when compared with MMP9-Low (Figure 4C: George et al.; Figure 4D: Jiang et al.). With the xCell algorithm, we found that, compared with MMP9-Low, MMP9-High has significant DCs and NKT (Figure 4E: George et al.; Figure 4F: Jiang et al.).

### MMP9 is Related to the Up-Regulation of the Immune-Related Signaling Pathway

**FIGURE 5:**
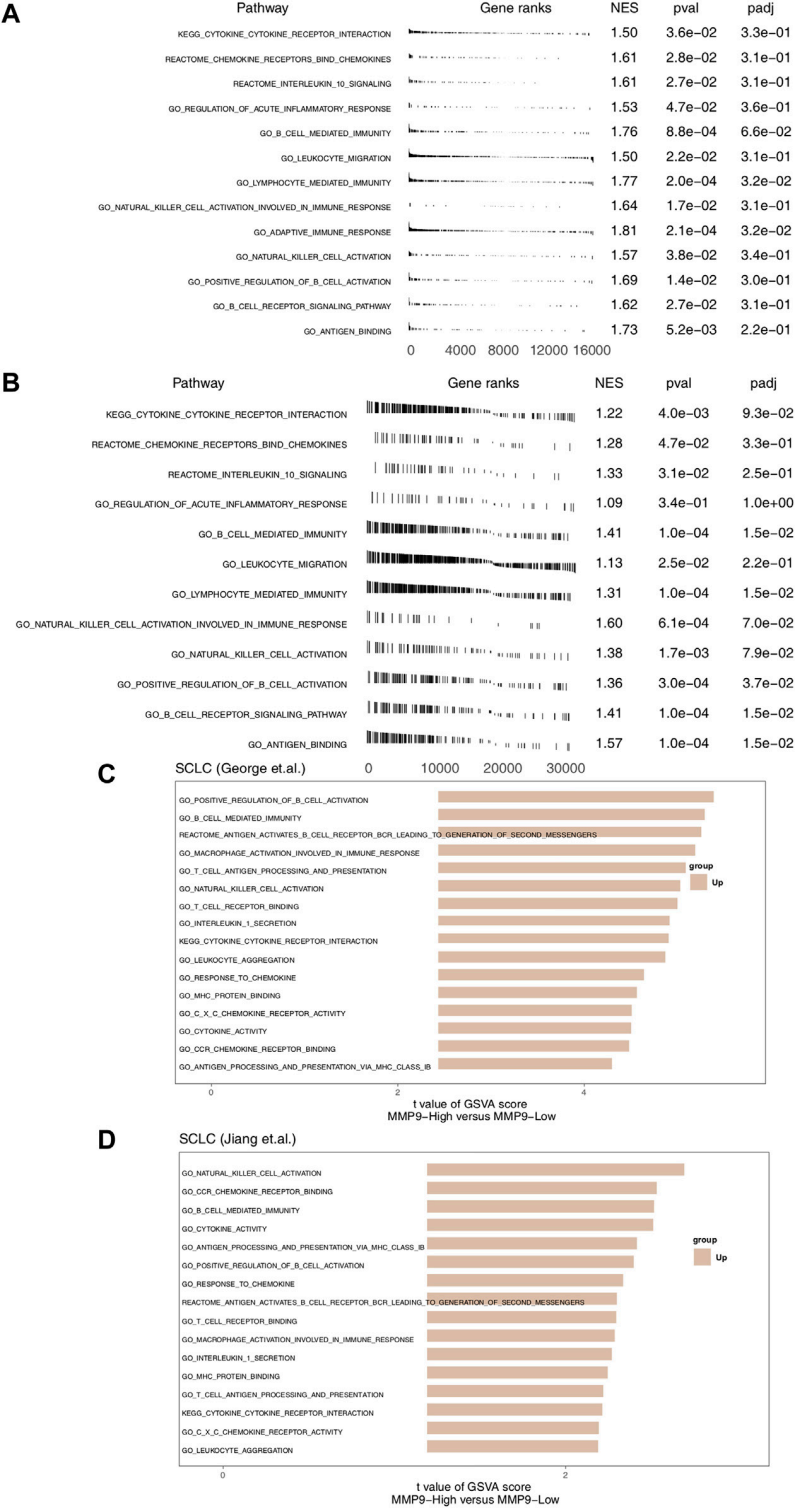
The association between MMP9 and immune-related signaling. **(A)** The results of GSEA in the SCLC (George et al.) relating to immune signaling. **(B)** The results of GSEA in the SCLC (Jiang et al.) relating to immune signaling. The GSEA of hallmark gene sets was downloaded from the MSigDB, and each run was performed with 1000 permutations. Differences in pathway activities scored per cell by MMP9-High and MMP9-Low tumors in the SCLC (George et al.) **(C)** and the SCLC (Jiang et al.) **(D)**. Shown are *t* values from a linear model.

In order to further explore the difference of signal pathway activity between MMP9-High and MMP9-Low, we used GSEA and ssGSEA to evaluate and calculate the signal pathway activity. Figures 5A,B shows that in SCLC (George et al.) and SCLC (Jiang et al.), MMP9-High has significantly increased activity of immune activation related pathways, such as cytokine binding and immune response mediated by B cells and NK cells when compared with MMP9-Low. In addition, the results of ssGSEA analysis showed that the activity of MMP9-High in B-cell, T-cell and NK cell activation, cytokine secretion, chemokine binding, and antigen presentation was significantly higher than that of MMP9-Low (Figures 5C,D).

### MMP9 is Related to the Prognosis of Immunotherapy

**FIGURE 6:**
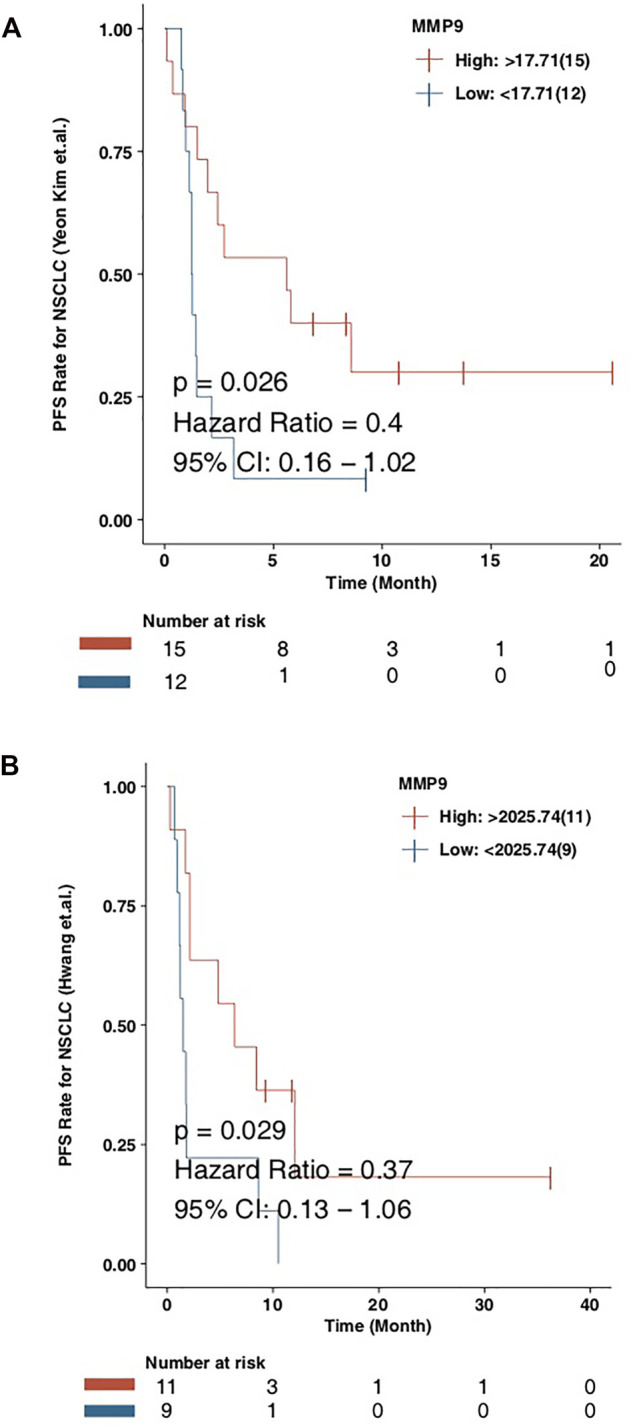
The prognostic value of the MMP9 in immunotherapy. Kaplan-Meier curves comparing the progress free survival (PFS) of patients with MMP9-High and patients with MMP9-Low in the NSCLC (Kim et al.) **(A)** and the NSCLC (Hwang et al.) **(B)**.

In order to explore the role of MMP9 in the prognosis of patients receiving immune checkpoint inhibitors, we used CAMOIP as a web tool to verify the relationship between MMP9 and the prognosis of immunotherapy. We found that in the NSCLC (Kim et al.) cohort, the PFS time of MMP9-High was significantly longer than that of MMP9-Low (Figure 6A; log-rank *p* = 0.026; HR = 0.4). In another NSCLC (Hwang et al.) cohort, the PFS time of MMP9-High was significantly longer than that of MMP9-Low (Figure 6B; log-rank *p* = 0.029; HR = 0.37). These results suggest that MMP9 may be an important biomarker for the prognosis of immunotherapy.

### Small Cell Lung Cancer Model Proves that MMP9 is Related to Cisplatin Sensitivity

**FIGURE 7:**
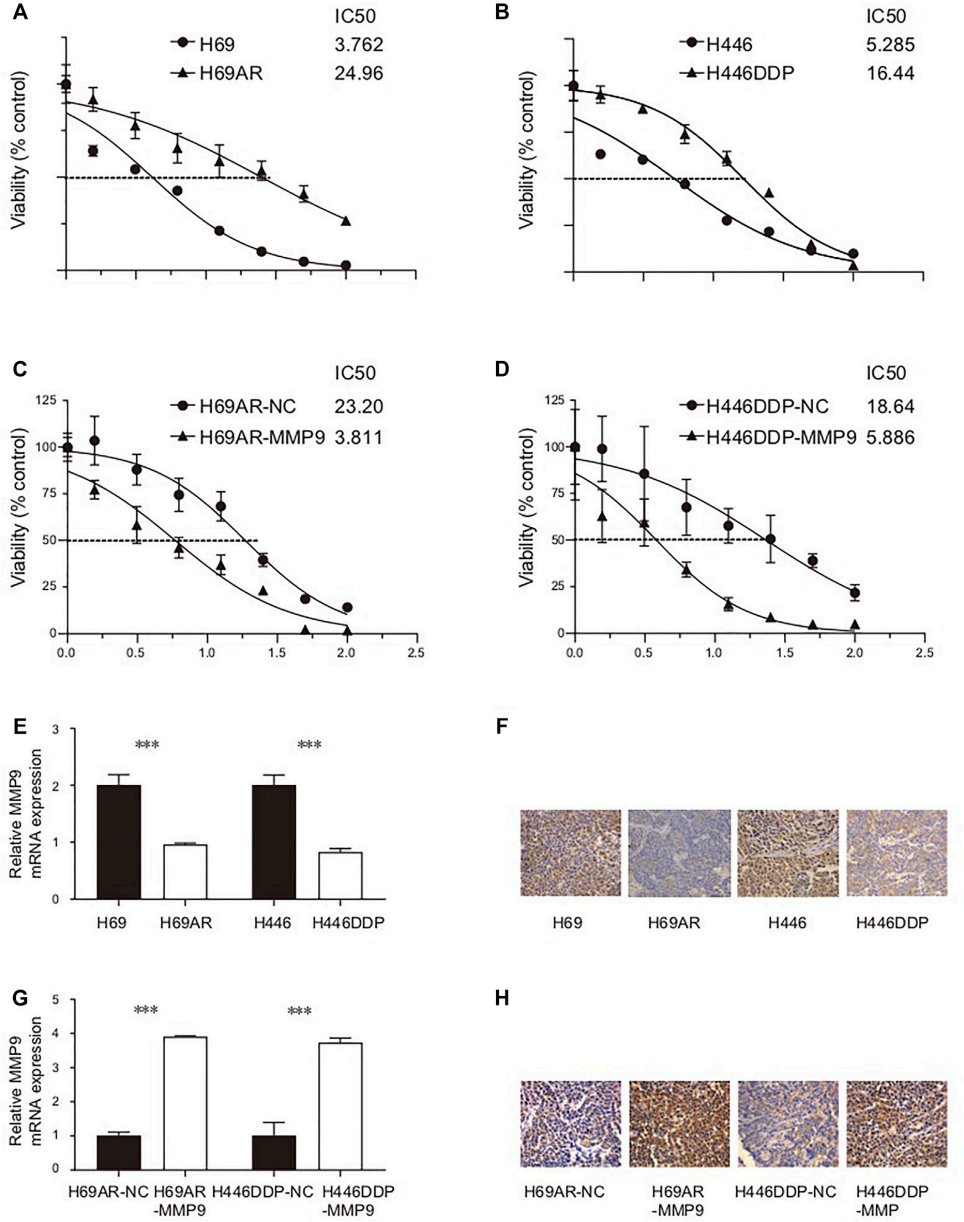
**(A)** The IC50 of H69/H69AR cells to cisplatin. **(B)** The IC50 of H446/H446DDP to cisplatin. **(C)** The IC50 of H69AR-NC/H69AR-MMP9 to cisplatin. **(D)** The IC50 of H446DDP-NC/H446DDP-MMP9 to cisplatin. **(E)** The expression level of MMP9 mRNA in H69, H69AR, H446 and H446DDP. **(F)** The expression level of CDYL protein in H69, H69AR, H446 and H446DDP. **(G)** The expression level of MMP9 mRNA in H69AR-NC, H69AR-MMP9, H446DDP-NC and H446DDP-MMP9. **(H)** The expression level of CDYL protein in H69AR-NC, H69AR-MMP9, H446DDP-NC and H446DDP-MMP9 (***p < 0.001).

In this study, the IC50 values of two pairs of chemotherapy-sensitive and drug-resistant small cell lung cancer cells to the first-line chemotherapy drug (cisplatin) were detected using the CCK8 method. The results showed that the IC50 of chemotherapy-sensitive H69/H446 small cell lung cancer cells to cisplatin was significantly lower than that of the corresponding chemotherapy-resistant cells (H69AR/H446DDP) (Figures 7A,B). To detect the effect of MMP9 on the chemotherapy resistance of small cell lung cancer cells, this study used the CCK8 method to detect the IC50 value of chemotherapy-resistant small cell lung cancer cells with upregulated expression of MMP9 to first-line chemotherapy drugs (cisplatin and etoposide). The results showed that upregulating the expression level of MMP9 (H69-MMP9, H446-MMP9) in chemotherapy-resistant small cell lung cancer cells significantly reduced the IC50 value of SCLC cells to chemotherapy drugs (Figures 7C,D), and the subsequent chemotherapy resistance of cells decreased.

Firstly, the basic expression levels of MMP9 mRNA and MMP9 protein in small cell lung cancer cells were detected by real-time fluorescence quantitative PCR and immunohistochemistry. The results of the real-time quantitative PCR showed that the expression level of MMP9 mRNA in chemotherapy-resistant H69AR cells and H446DDP cells was significantly lower than that in chemotherapy-sensitive H69 cells and H446 cells (Figure 7E) (*p* < 0.001). Immunohistochemically, the results also showed that the expression level of CDYL protein in chemotherapy-resistant H69AR cells and H446DDP cells was lower than that of their parents’ chemotherapy-sensitive H69 cells and H446 cells (Figure 7F). Based on the basic expression level of MMP9 in the above two pairs of chemotherapy-sensitive and chemotherapy-resistant small cell lung cancer cells, this study further uses lentivirus-mediated LV5-MMP9 to up-regulate the expression level of MMP9 in chemotherapy-resistant H69AR and H446DDP small cell lung cancer cells. The verification results of quantitative PCR, and immunohistochemistry showed that we successfully constructed the small cell lung cancer cell model with an up-regulated expression of CDYL (H69AR-MMP9, H446DDP-MMP9). (Figures 7G,H). (*p* < 0.001).

## Discussion

The results of the univariate COX regression, multivariate COX regression, and KM analysis demonstrated that MMP9-High may be an independent predictor of improved prognosis in SCLC patients after receiving cisplatin. In addition, the expression of MMP9 was negatively correlated with the IC50 value of cisplatin. Based on the observations pertaining to MMP9, we analyzed the TIME of patients with SCLC. Compared with MMP9-Low, MMP9-High displayed significantly increased activated immune cells and an amplified active immune activation pathway. In addition, according to the immunotherapy cohort, MMP9 may represent a suitable novel biomarker for screening patients undergoing immunotherapy.

Remodeling the immunogenicity of tumor cells may be one of the reasons underlying the improved prognosis of patients with MMP9-High SCLC after cisplatin treatment. After cisplatin induces the death of tumor cells, it will release immunogenic substances originally located in tumor cells, thus activating the APC-mediated antigen presentation process, resulting in an anti-tumor immune response. Evidence from a study investigating the anti-tumor mechanism of cisplatin employed protein omics based on mass spectrometry in order to detect the content of protein in the supernatant of tumor cell culture before and after cisplatin administration. A total of 2,239 varieties of protein were identified, of which 526 types were up-regulated more than 3 times after cisplatin treatment, including tumor-driving genes such as NRAS, heat shock proteins, metabolic enzymes, and other proteins. Furthermore, APC stimulated by antigenic substances in these supernatants can significantly enhance the proliferation and functional level of CD8+T cells, suggesting that antigen release induced by chemotherapeutic drugs can significantly activate anti-tumor immune response through the antigen presentation system (Beyranvand Nejad et al., 2016; Lin et al., 2020).

In this study, we found that MMP9-High has significantly higher TMB and NALs than MMP9-Low. Up-regulation of immunocompetent ligand on the surface of tumor cells may contribute to the possible mechanisms of improved prognosis in patients with MMP9-High SCLC after cisplatin treatment. In addition to the above-mentioned release of immunogenic substances related to cell death, chemotherapeutic drugs can also affect the protein expression of tumor cells. Immunogenic tumor cells remodeling tumor cells can down-regulate the expression of MHCI to avoid the killing effect of CTL cells. It has been found that cisplatin can up-regulate MHCI on the surface of head and neck cancer cells and enhance the presentation of tumor antigens, thus promoting the recognition of tumor cells by CTL and the activation of CD8+T cells (Gameiro et al., 2012; Tran et al., 2017; Lin et al., 2021b). In addition, cisplatin can also lead to the up-regulation of MHCI expression in ovarian cancer cells (Grabosch et al., 2019). After subcutaneous inoculation of tumor cells pretreated with cisplatin *in vitro*, it was found that, when compared with the control group, the tumor cells treated with cisplatin were not likely to form tumors, and the expression of MHCI in grown tumors was higher (Nio et al., 2000). Similar results were obtained with regard to colon cancer cells (Ohtsukasa et al., 2003). We can infer that cisplatin can up-regulate the expression of MHCI *in vitro* and *in vivo*. In addition to tumor cells, chemotherapeutics can up-regulate the expression level of MHCI in antigen presenting cells (APCs) (Jackaman et al., 2012). In addition, studies reveal that cisplatin can improve the antigen presenting ability of APC such as DCs(Shurin et al., 2009; Zitvogel et al., 2013; Lin et al., 2021c). In this study, we found that MMP9-High has significantly infiltrated DCs, higher MHC, and increased antigen presentation activities compared to that of MMP9-Low.

Inflammatory TIME may be one of the reasons for the improved prognosis of patients with MMP9-High SCLC after cisplatin treatment. Chemotherapy drugs can enhance the sensitivity of tumor cells to immune killing. For example, cisplatin can enhance the sensitivity of tumor cells to the CTL specific killing effect (Ramakrishnan et al., 2012). In addition, active immune effector cells, such as NKs, serve to mediate cytotoxicity (Lichtenstein and Pende, 1986). Furthermore, cisplatin also causes effector cells to produce more cytokines that regulate and promote various immune responses (Kepp et al., 2013; Huang et al., 2021). Moreover, chemotherapy can eliminate immunosuppressive cells such as MDSC and Tregs by inducing apoptosis and through other mechanisms, so that immunotherapy can achieve the maximum efficacy. For example, the use of cisplatin before the injection of DNA vaccine encoding CRT can reduce the level of MDSC in tumor-bearing mice (Tseng et al., 2008; Li et al., 2020). Cisplatin is beneficial in inducing the formation of self-reactive T-cells and anti-tumor immune responses (Tseng et al., 2008). The CTLs and NKs with significant infiltration in MMP9-High, respectively, verified the above results.

However, this research has some limitations. First, the cohorts of SCLC are very limited, and this study only includes two of the SCLC cohorts recorded in the current public data. Secondly, the SCLC cohort lacks data on cisplatin drug sensitivity, and the data on cisplatin drug sensitivity in this study is based on the pRRophetic algorithm.

## Conclusion

Based on the results obtained in this study, we identified that MMP9-High may be a potential new biomarker that facilitates the improved prognosis of SCLC patients after cisplatin treatment. In addition, the TIME of MMP9-High SCLC is primarily characterized by a large number of infiltrated activated immune cells and activated immune-related pathways.

## Data Availability

The original contributions presented in the study are included in the article/Supplementary Material, further inquiries can be directed to the corresponding authors.
