# Supplementary material for: MMP9 Expression Correlates With Cisplatin Resistance in Small Cell Lung Cancer Patients
Source: Front Pharmacol. 2022 Apr 1;13:868203. doi: 10.3389/fphar.2022.868203 (PMC9010875; doi:10.3389/fphar.2022.868203)
Supplement: Supplementary file 1 [file DataSheet2.PDF]

| | High (N=24) | Low (N=24) | Overall (N=48) |
|---|---|---|---|
| **Biopsy** | | | |
| Chemo | 4 (16.7%) | 4 (16.7%) | 8 (16.7%) |
| Naive | 20 (83.3%) | 20 (83.3%) | 40 (83.3%) |
| **Age** | | | |
| Mean (SD) | 58.5 (8.82) | 55.9 (8.29) | 57.2 (8.56) |
| Median [Min, Max] | 61.0 [36.0, 70.0] | 55.0 [39.4, 71.0] | 57.3 [36.0, 71.0] |
