# Supplementary material for: MMP9 Expression Correlates With Cisplatin Resistance in Small Cell Lung Cancer Patients
Source: Front Pharmacol. 2022 Apr 1;13:868203. doi: 10.3389/fphar.2022.868203 (PMC9010875; doi:10.3389/fphar.2022.868203)
Supplement: Supplementary file 2 [file DataSheet1.PDF]

	High (N=34)	Low (N=34)	Overall (N=68)
**Gender**			
Female	13 (38.2%)	7 (20.6%)	20 (29.4%)
Male	21 (61.8%)	27 (79.4%)	48 (70.6%)
**Ethnicity**			
Asian	5 (14.7%)	3 (8.8%)	8 (11.8%)
Caucasian	13 (38.2%)	17 (50.0%)	30 (44.1%)
Missing	16 (47.1%)	14 (41.2%)	30 (44.1%)
**Stage**			
I	12 (35.3%)	15 (44.1%)	27 (39.7%)
II	7 (20.6%)	7 (20.6%)	14 (20.6%)
III	10 (29.4%)	9 (26.5%)	19 (27.9%)
IV	5 (14.7%)	3 (8.8%)	8 (11.8%)
**Smoker**			
Current	18 (52.9%)	19 (55.9%)	37 (54.4%)
Former	13 (38.2%)	15 (44.1%)	28 (41.2%)
Never	2 (5.9%)	0 (0%)	2 (2.9%)
Missing	1 (2.9%)	0 (0%)	1 (1.5%)
**Pack Years**			
Mean (SD)	51.1 (28.8)	38.0 (20.5)	44.4 (25.5)
Median [Min, Max]	50.0 [0, 100]	40.0 [0.750, 90.0]	45.0 [0, 100]
Missing	11 (32.4%)	10 (29.4%)	21 (30.9%)
**Age**			
Mean (SD)	64.1 (8.40)	64.9 (9.43)	64.5 (8.87)
Median [Min, Max]	64.5 [47.0, 82.0]	64.0 [47.0, 83.0]	64.0 [47.0, 83.0]
