# Supplementary figures and images for: MMP9 Expression Correlates With Cisplatin Resistance in Small Cell Lung Cancer Patients
Source: Front Pharmacol. 2022 Apr 1;13:868203. doi: 10.3389/fphar.2022.868203 (PMC9010875; doi:10.3389/fphar.2022.868203)

**A**

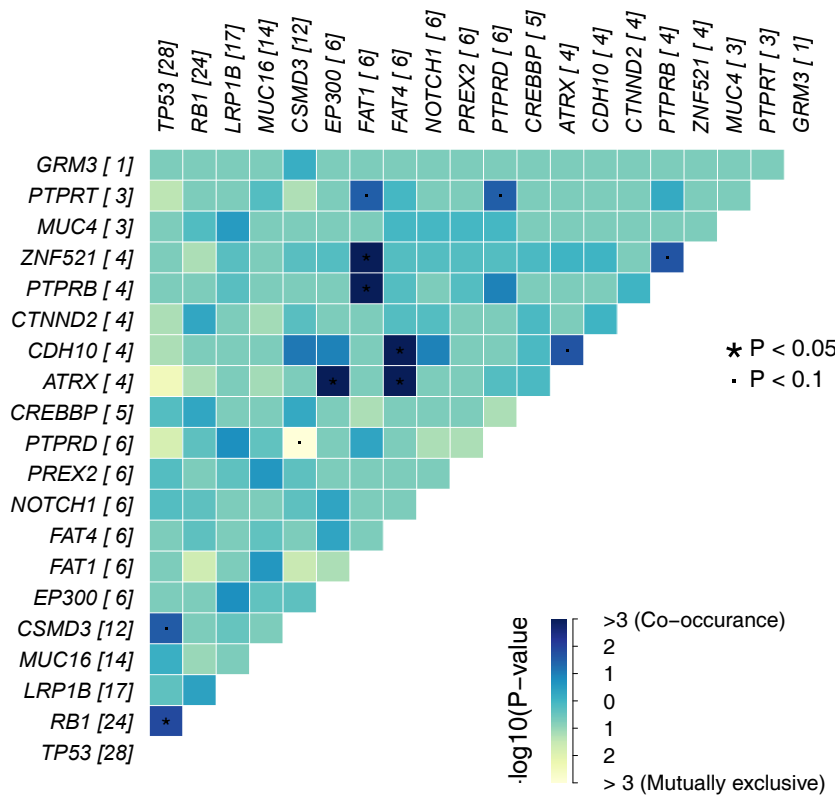

**B**

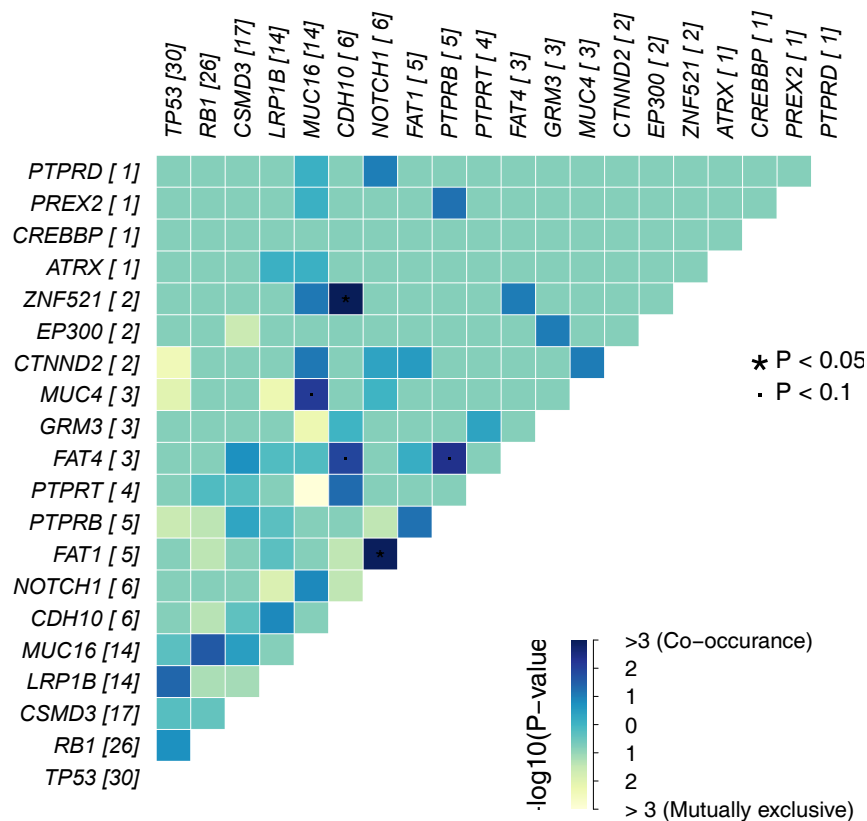

Supplement: Supplementary file 3 [file Image1.PDF]
